# Three-dimensional analysis of interstitial cells in the lamina propria of the murine vas deferens by confocal laser scanning microscopy and FIB/SEM

**DOI:** 10.1038/s41598-022-13245-7

**Published:** 2022-06-08

**Authors:** Tasuku Hiroshige, Kei-Ichiro Uemura, Shingo Hirashima, Akinobu Togo, Keisuke Ohta, Kei-Ichiro Nakamura, Tsukasa Igawa

**Affiliations:** 1 Department of Urology, Kurume University School of Medicine, Kurume, 830-0011 Japan; 2 Division Microscopic and Development Anatomy, Department of Anatomy, Kurume University School of Medicine, Kurume, 830-0011 Japan; 3 Advanced Imaging Research Center, Kurume University School of Medicine, Kurume, 830-0011 Japan; 4 Cognitive and Molecular Research Institute of Brain Diseases, Kurume University School of Medicine, Kurume, 830-0011 Japan

**Keywords:** Electron microscopy, Cell biology, Urology

## Abstract

The present study aimed to explore the three-dimensional (3D) ultrastructure of interstitial cells (ICs) within the lamina propria of the murine vas deferens and the spatial relationships between epithelial cells and surrounding cells. Focused ion beam scanning electron microscopy and confocal laser scanning microscopy were performed. ICs within the lamina propria had a flat, sheet-like structure of cytoplasm with multiple cellular processes. In addition, two types of 3D structures that comprised cell processes of flat, sheet-like ICs were observed: one was an accordion fold-like structure and the other was a rod-shaped structure. ICs were located parallel to the epithelium and were connected to each other via gap junctions or adherens junctions. Moreover, multiple sphere-shaped extracellular vesicle-like structures were frequently observed around the ICs. The ICs formed a complex 3D network comprising sheet-like cytoplasm and multiple cell processes with different 3D structures. From this morphological study, we noted that ICs within the lamina propria of murine vas deferens may be involved in signal transmission between the epithelium and smooth muscle cells by physical interaction and by exchanging extracellular vesicles.

## Introduction

The vas deferens, which is composed of a muscle coat, inner mucosa, and outer adventitia, is a pair of tiny muscular tubes of the male reproductive system that connects the epididymis to the ejaculatory duct. The vas deferens contracts rhythmically to transport sperm from the epididymis to the ejaculatory ducts and strongly contracts to release the stored sperm during ejaculation^1,2^. Therefore, a deeper understanding of the contraction functions of the vas deferens is needed for better elucidation of the ejaculation mechanism.

The contractility of smooth muscles is regulated by the surrounding cells, including neurons and surrounding vasculature and endothelial cells, and many studies have detailed the underlying intercellular mechanisms^3^. In addition, recent studies in several smooth muscle organs, including the vas deferens, suggest that the epithelium modulates the contractility of smooth muscles^4–6^; however, the mechanisms underlying this regulation remain unknown.

Interstitial cells (ICs) have been reported to be involved in numerous physiological functions, including structural support, neurotransmission, intercellular communication, and neo-angiogenesis regeneration^7,8^. In various smooth muscle organs, ICs have been reported to play an important role in regulating smooth muscle movements^9–11^. In addition, ICs have different immunohistochemical, morphological, and functional differences depending on their localization^12–14^. ICs within the lamina propria in the bladder may be involved in signal transmission between the epithelium and smooth muscles^15^.

Many morphological studies on ICs have been reported as the first step in clarifying the functional role of various ICs due to their characteristic morphology of multiple, highly elongated processes^16–19^. Understanding the three-dimensional (3D) structure is important for more accurate morphological evaluation; however, it is difficult to understand the 3D structure and spatial relationship between ICs and surrounding tissues using two-dimensional (2D) observation of sections due to its complex morphology.

Focused ion beam/scanning electron microscopy (FIB/SEM) tomography is a novel approach for 3D imaging and quantitative analysis of nano- and meso-scale morphologies using serial milling with nanometer steps and image acquisition^20^. Reconstructing the 3D appearance of ICs from a set of 2D images taken by FIB/SEM tomography allowed the extraction of valuable data regarding 3D morphology in mesoscale dimensions, which is unobtainable by conventional 2D observation^21^.

In the present study, using 3D morphological analysis via confocal laser scanning microscopy (CLSM) and FIB/SEM, we observed the 3D fine structures of ICs within the lamina propria of the murine vas deferens and analyzed the spatial relationships between ICs and surrounding tissues, including the epithelium, as the first step to determine whether ICs are involves in signal transmission from the epithelium to smooth muscles.

## Methods

### Ethics consideration

All experiments were performed in accordance with the National Institutes of Health Guidelines for Animal Research and Animal Research: Reporting of In Vivo Experiments (ARRIVE) guidelines (https://www.nc3rs.org.uk/arrive-guidelines). All animal procedures were approved by the Board for Animal Experiments at the Kurume University School of Medicine, Kurume, Japan.

### Preparation of specimens for light microscopy.

Twelve-week-old male C57BL/6 mice (n = 3) were euthanized using combined anesthesia, composed of 0.3 mg/kg medetomidine, 4.0 mg/kg midazolam, and 5.0 mg/kg butorphanol. The mouse tissues were fixed with 4% paraformaldehyde in phosphate-buffered saline (PBS). After perfusion fixation, the abdomen was opened by a median incision to extract the bilateral pars vas deferens. The extracted tissue samples were immersed in the same fixative for 2 h at 4 °C. Then, the samples were trimmed, washed three times for 5 min in PBS, immersed in PBS containing 30% sucrose overnight at 4 °C, and frozen in the Tissue-Tek® O.C.T compound (Sakura Finetek, Torrance, CA, USA).

### Preparation of specimens for FIB/SEM

The specimens for FIB/SEM were prepared as described previously^22^. The mice (n = 3) were euthanized as described above, and the mouse tissues were fixed with half-strength Karnovsky solution [2% paraformaldehyde and placement in 2.5% glutaraldehyde in 0.1 M cacodylate buffer (pH 7.3)]. The extracted tissue samples were immersed in the same fixative for 2 h at 4 °C and rinsed in buffer three times for 10 min each. Next, the specimens were cut into small cubes and subjected to post-fixation treatment and *en bloc* staining as follows: after three washes in cacodylate buffer, the specimens were post-fixed for 2 h in a solution containing 2% osmium tetroxide and 1.5% potassium ferrocyanide in cacodylate buffer at 4 °C. The specimens were then washed three times with distilled water and immersed in a 1% thiocarbohydrazide solution for 1 h. After five washes with distilled water, the specimens were immersed in 2% osmium tetroxide in distilled water and washed three times with distilled water. The specimens were then stained *en bloc* in a solution containing 4% uranyl acetate dissolved in distilled water overnight for contrast enhancement, followed by a distilled water wash. Next, the specimens were stained with Walton’s lead aspartate solution for 1 h^23^. Subsequently, the specimens were dehydrated using an ethanol series (25%, 50%, 70%, 80%, 90%, and twice in 100% for 10 min each), followed by infiltration with an epoxy resin mixture (Epon 812, TAAB, Barks, England) and polymerization for 72 h at 60 °C. To expose the cross-sections, the surfaces of the embedded specimens were cut vertically, using a diamond knife with an Ultracut E Microtome (Leica, Wetzlar, Germany). The resin blocks were then placed in the specimen holder of a standard SEM with adhesives for imaging.

### Immunohistochemistry

We have previously reported the following protocols of the present study^24^. Cryosections with a thickness of 50 μm were prepared using a CM1950 cryomicrotome (Leica, Wetzlar, Germany) and immersed in PBS. The floating sections were blocked via treatment with 3% normal goat serum, 3% normal donkey serum, and 0.5% Triton X-100 in PBS for 60 min, followed by treatment with the primary antibody diluted in blocking solution overnight at 4 °C (Supplementary Table S1). This was followed by rinsing three times with PBS. The sections were then treated with a secondary antibody for 1 h (1:500 dilution) at room temperature (22 °C–25 °C). Alexa Fluor-488-conjugated donkey anti-goat IgG, Alexa Fluor-568-conjugated donkey anti-rat IgG, and Alexa Fluor-568-conjugated donkey anti-rabbit IgG (1:500 dilution; Invitrogen, Waltham, MA, USA) were used as secondary antibodies. After rinsing with PBS, the sections were mounted using PermaFluor mounting medium (Thermo Fischer Scientific, Shandon, PA, USA) and observed under a CLSM (FV1000, Olympus, Tokyo, Japan) with the following acquisition parameters: excitation at 473 and 559 nm, 60× oil immersion lens (NA = 1.2), and image size = 105 × 105 μm. The Z-stack sizes varied between 50 and 80 images. Image deconvolution and 3D reconstruction were performed using the resulting image stacks analyzed using Avizo 9.1.1 (FEI, Burlington, MA, USA).

### FIB/SEM tomography and 3D structure reconstruction

FIB/SEM tomography analysis was performed as described previously^24^. Freshly exposed surfaces of the specimens were examined using backscatter electron imaging with a conventional field emission SEM with FIB (FIB/SEM, Quanta Three-dimensional FEG, FEI, Eindhoven, The Netherlands). To prevent charging, the specimens were coated with a thin layer of evaporated osmium^25^. Serial images of the block face were acquired through repeated cycles of sample surface milling and imaging using the Slice & View G2 operating software (FEI). Milling was performed using a gallium ion beam at 30 kV with a current of 15 mA, and the milling pitch was set to 50 nm/step and 1500 cycles. Images were acquired at a landing energy of 3 keV with a bias voltage of 1.5 kV. The resultant image stack was processed using ImageJ (https://imagej.nih.gov/ij/) and Avizo 9.1.1 (FEI, Burlington, MA, USA). Avizo is a user-friendly application that allows for 2D images, slices known as orthos, to be digitally analyzed, segmented, color-coded, and rendered for 3D reconstruction. To observe the 3D structures of the ICs, the components of the cell membrane were semi-automatically segmented. Subsequently, the cells were visualized, and images were displayed.

## Results

### Immunohistochemistry

**Figure 1:**
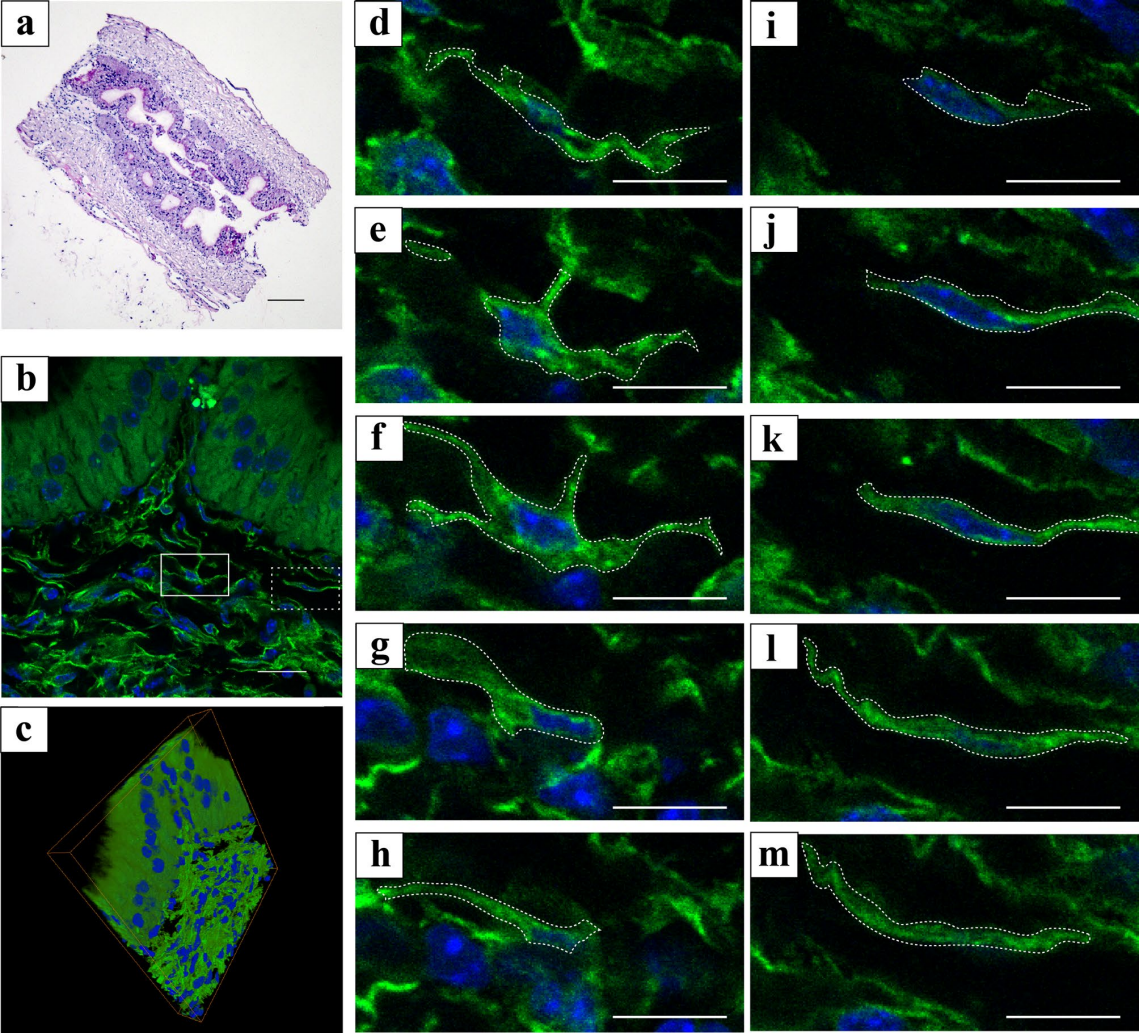
(**a**) Hematoxylin and Eosin-stained sagittal section of the murine vas deferens. (**b**) 2D digital slice extracted from the sequential immunofluorescence images for platelet-derived growth factor α (PDGFRα) (green). (**c**) Oblique view of reconstructed sequential 3D immunofluorescence images of PDGFRα (green). (**d**–**h**) Sequential high-magnification immunofluorescence images of white line square area in (**b**). (**i**–**m**) Sequential high-magnification immunofluorescence images of white dot square area in (**b**). PDGFRα-immunoreactive areas were shown by white dotted line box. Nuclei were counterstained in blue with 4′,6-diamidino-2-phenylindole (**b**–**m**). The images underwent deconvolution and 3D reconstruction using the Avizo software (version 9.1.1). Scale bar: 200 µm (**a**), 20 µm (**d**–**m**).

Z-stack immunostaining images of 50-μm sections depicted the 3D structure of the platelet-derived growth factor α (PDGFRα) immunoreactive area under CLSM after observing Hematoxylin–Eosin stained sagittal sections of the vas deferens (Fig. 1). Multiple PDGFRα-immunoreactive areas were observed within the lamina propria in the 2D digital slice extracted from the sequential images (Fig. 1b); these areas are laid on top of one another, like a wall in parallel to the epithelium, in the 3D image stack (Fig. 1c). In the high-magnification sequential images of the PDGFRα immunoreactive areas, two types of PDGFRα-immunoreactive areas were observed: one with multipolar processes (white dotted line box, Fig. 1d–h) and one with bipolar processes (white dotted line box, Fig. 1i–m).

**Figure 2:**
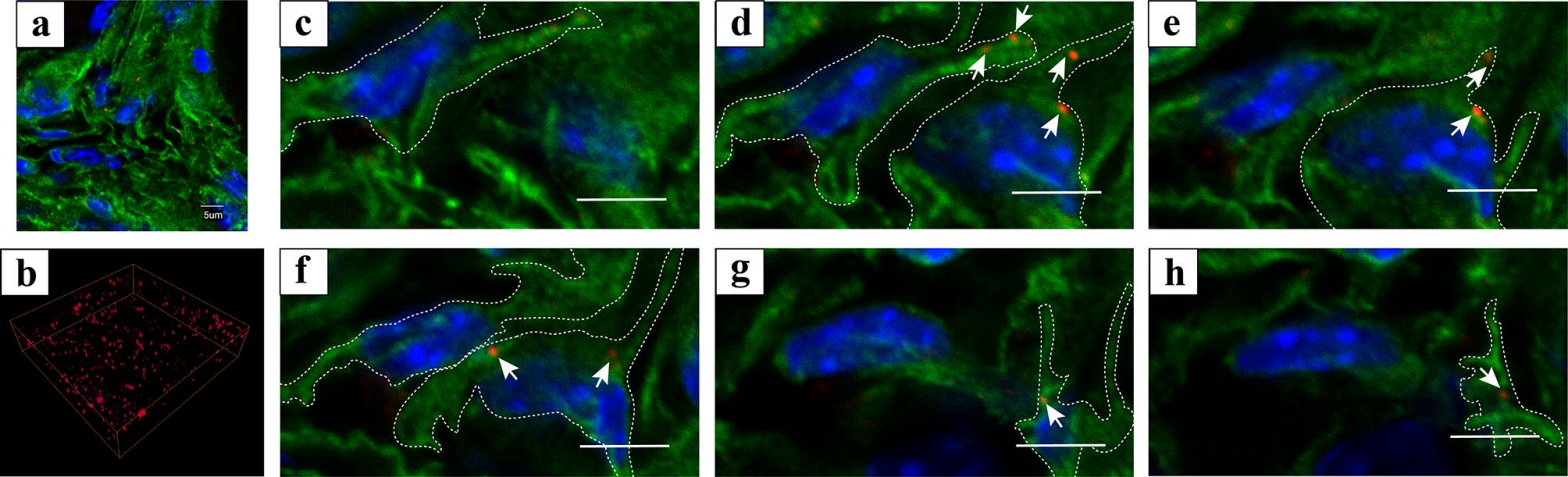
(**a**) 2D digital slice extracted from the sequential immunofluorescence images for platelet-derived growth factor α (PDGFRα) (green) and connexin 43 (red). (**b**) Reconstructed sequential 2D digital slice immunofluorescence images for connexin 43 (red). (**c**–**h**) Sequential high-magnification immunofluorescence images of frozen sections for PDGFRα (green) and connexin 43 (red). Connexin43 immunoreactive areas were shown by white arrows and PDGFRα-immunoreactive areas including connexin43 immunoreactive areas were shown by white dotted line box. Nuclei were counterstained in blue with 4′,6-diamidino-2-phenylindole (**a**, **c**–**h**). The images underwent deconvolution and 3D reconstruction using the Avizo software (version 9.1.1). Scale bar: 20 µm (**c**–**h**).

Double immunostaining for connexin43 and PDGFRα was performed to investigate whether PDGFRα-positive cells had gap junctions. A small number of punctate connexin43 immunoreactive areas were observed within PDGFRα-immunoreactive areas in the 2D digital slice extracted from the sequential images (Fig. 2a); however, many punctate connexin43 immunoreactive areas were observed within the 3D image stack (Fig. 2b). In the high-magnification sequential images, most connexin43 immunoreactive areas (white arrows in Fig. 2c–h) were observed within PDGFRα immunoreactive areas (white dotted line box, in Fig. 2c–h).

In the double immunostaining for ionized calcium-binding adaptor molecule 1 (Iba-1) and PDGFRα, macrophages labeled with Iba-1 were observed in the 2D digital slice extracted from the sequential images (Supplementary Fig. S1a). In the 3D image stack, many elongated cell processes of macrophages were observed within the lamina propria (Supplementary Fig. S1b). Macrophages were in close proximity to the PDGFRα-immunoreactive areas over several 2D digital slices extracted from sequential images (Supplementary Fig. S1c–h). In the double immunostaining for β-3tubulin and PDGFRα, nerves labeled with β-3tubulin were observed in the 2D digital slice extracted from the sequential images (Supplementary Fig. S2a). In the 3D image stack, many nerves were observed within the lamina propria (Supplementary Fig. S2b). Nerves were also in close proximity to PDGFRα-immunoreactive areas over several 2D digital slices extracted from sequential images (Supplementary Fig. S2c–h).

### FIB/SEM tomography

**Figure 3:**
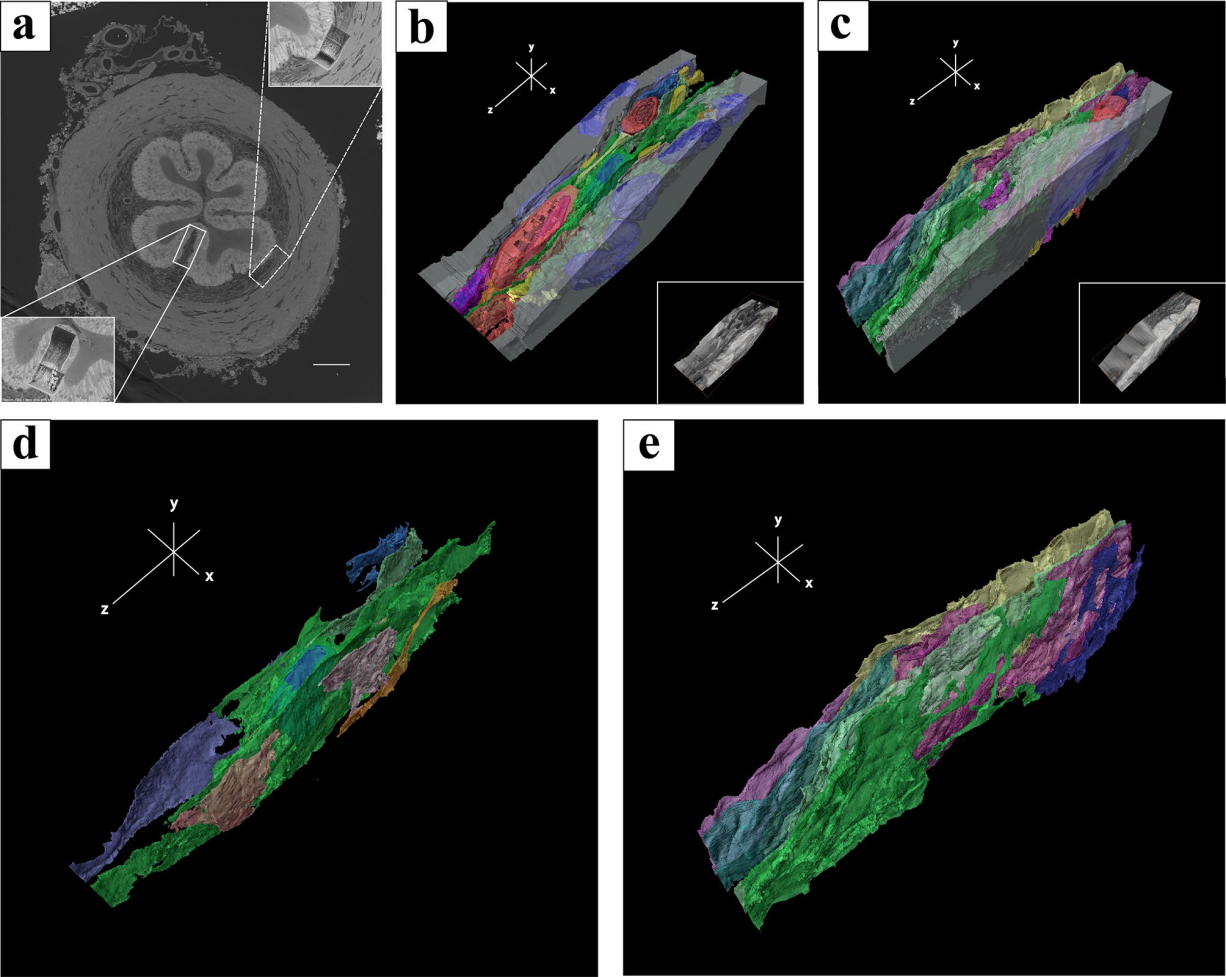
(**a**) Surface observation of the murine vas deferens using focused ion beam/scanning electron microscopy (FIB/SEM). The high magnification area in the lamina propria after a series of sequential images were acquired using FIB/SEM [lower left inset of (**a**), upper right inset of (**a**)]. (**b**) 3D-reconstruction of interstitial cells (ICs) surrounding tissue and cells within the lamina propria in lower left inset of (**a**). Tissue block reconstructed from 1,500 FIB/SEM images with 50 nm spacing in the longitudinal muscle layer at a pixel size of 14.5703 × 14.5703 nm^2^ [lower right inset of (**b**)]. (**c**) 3D-reconstruction of ICs surrounding tissue and cells within the lamina propria in upper right inset of (**a**). Tissue block reconstructed from 1,100 FIB/SEM images with 50 nm spacing in the orbital muscle layer at a pixel size of 9.10645 × 9.10645 × 50 nm^2^ [lower right inset of (**c**)]. (**d**) 3D-reconstruction of ICs extracted from (**b**). (**e**) 3D-reconstruction of ICs extracted from (**c**). The images underwent deconvolution and 3D reconstruction using the Avizo software (version 9.1.1).

Two image stacks were obtained from each mouse (Fig. 3a). One image stack was obtained within the lamina propria beneath the epithelium (14.5703 × 14.5703 × 50 nm^3^ voxel size, 1,500 serial slices) (inset of Fig. 3b), and the other image stack was obtained within the lamina propria between epithelial folds (9.10645 × 9.10645 × 50 nm^3^ voxel size, 1,100 serial slices) (inset of Fig. 3c). All structures including ICs and surrounding tissues in the image stacks were reconstructed (Fig. 3b, c), where reconstructed ICs were 16 in total. The 3D reconstruction of ICs involved sheet-like structures and laid on top of one another in both image stacks (Fig. 3d, e).

**Figure 4:**
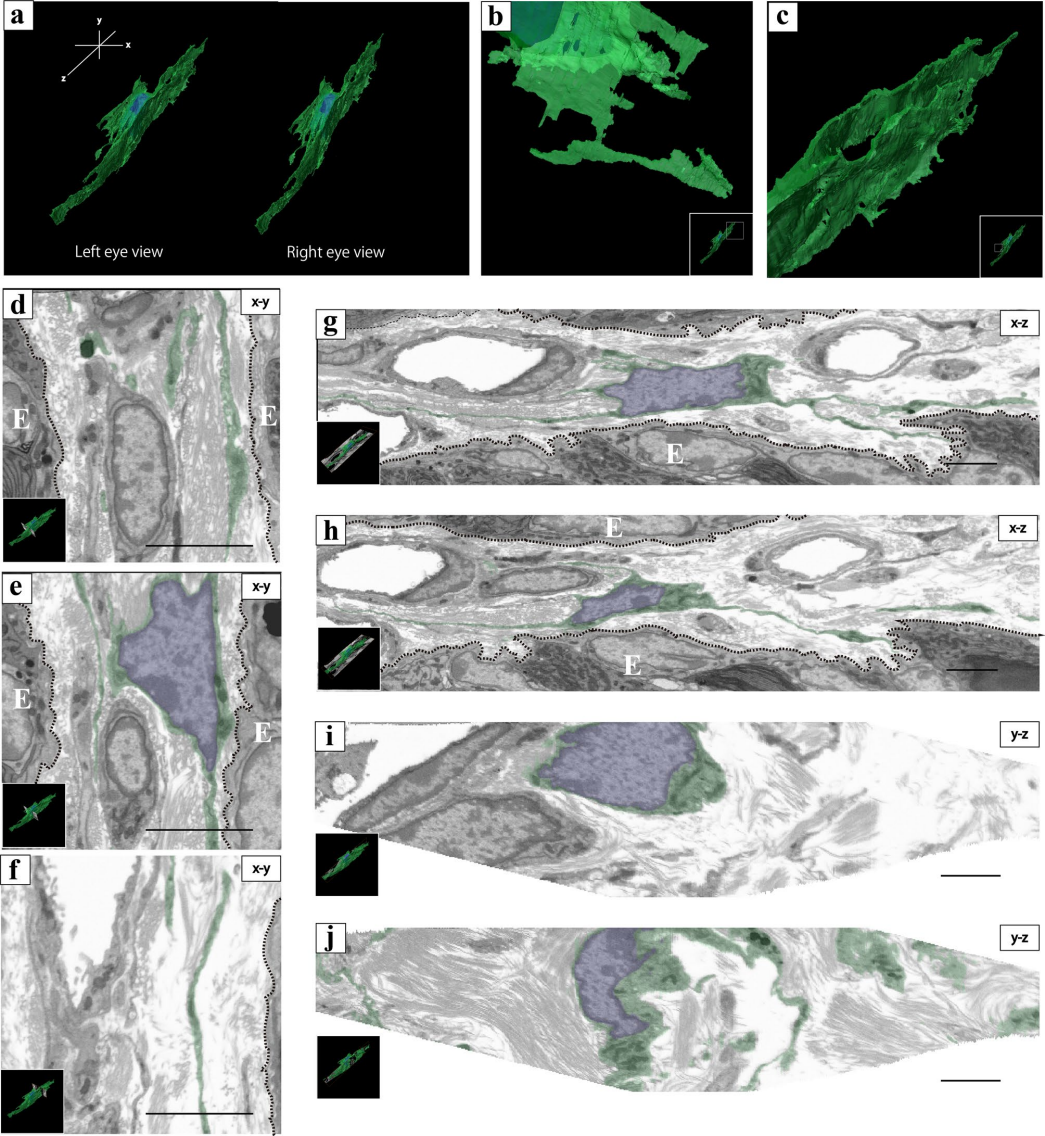
(**a**) Stereoscopic 3D reconstructed image of the flattened, sheet-like interstitial cell (IC). (**b**) A flattened, sheet-like shape of the IC. High magnification of the whole 3D reconstruction images [white line square area of inset of (**b**)]. (**c**) An elongated rod-like shape of the IC; high magnification of the whole 3D reconstruction images [white line square area of inset of (**c**)]. (**b**–**d**) 3D digital slices extracted from the sequential images of the IC along the XY direction. (**e**, **f**) 2D digital slices are extracted from the sequential images of the IC along the XZ direction. (**g**, **h**) 2D digital slices extracted from the sequential images of the IC along the YZ direction. The spatial relationship between 3D reconstructed image of the IC and 2D digital slices [inset of (**b–h**)]; E indicates the epithelium and epithelial basement membrane was shown by white dotted line box. For better visualization, the object surfaces are color-coded; IC, transparent green; nuclei, transparent blue. The images underwent deconvolution and 3D reconstruction using the Avizo software (version 9.1.1). Scale bar: 2 µm (**b**–**h**).

Of the reconstructed ICs, which had a sheet-like structure, two types of cell processes were observed (Fig. 4a): one was an elongated rod-like structure (Fig. 4b) and the other was folded cytoplasm like an accordion (Fig. 4c). Some 2D images in the XY direction showed that elongated narrow processes separated from the perinuclear cytoplasm were parallel to the epithelium (Fig. 4d–f). In the XZ direction, multiple elongated cell processes extending from the perinuclear cytoplasm were observed parallel to the epithelium. Some cell processes were observed over many sequential 2D slices (Fig. 4g, h), and other cell processes were observed over a few sequential 2D slices (Fig. 4g). 2D images in the YZ direction showed multiple island-like parts of the cytoplasm separated from the perinuclear cytoplasm and curved cell processes extending from the perinuclear cytoplasm (Fig. 4j). A small amount of cytoplasm was observed around the nucleus in the 2D sequential images in all directions (Fig. 4e, g–j).

**Figure 5:**
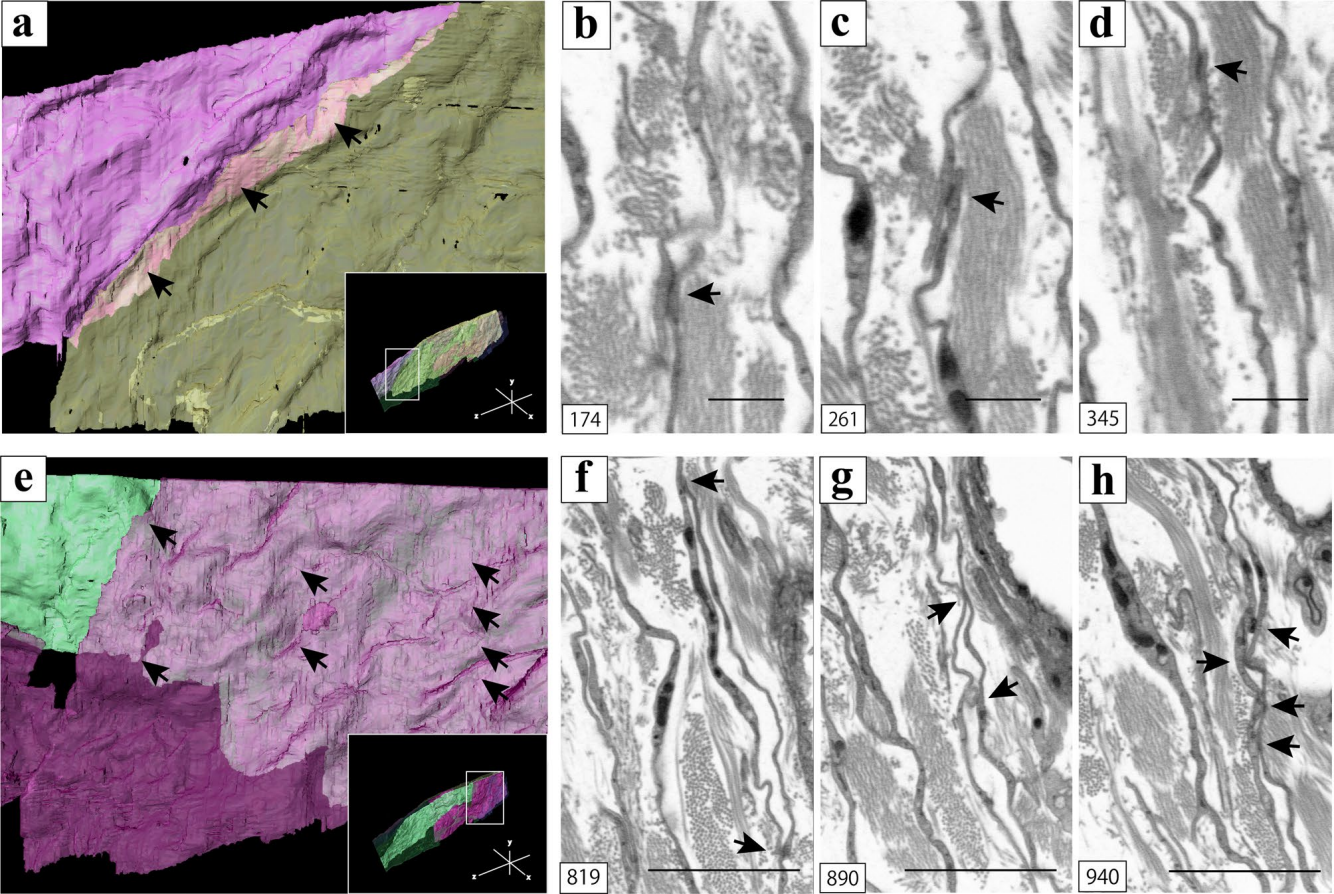
3D reconstruction of the proximity areas between interstitial cells (ICs) [black arrows of (**a**, **e**)]; high magnification of the whole 3D reconstruction images [white line square area of inset of (**a, e**)]. 2D digital slices including the area indicated by black arrows in (**a**) extracted from the sequential images. (**b**–**d**) 2D digital slices including the area indicated by black arrows in (**e**) extracted from the sequential images. (**f–h**) High-electron density was observed in the area indicated by black arrows in (**a**, **e**) [black arrows of (**b**–**d**, **f–h**)]. For better visualization, the object surfaces are color-coded; ICs, pink, yellow, light green, and purple. The images underwent deconvolution and 3D reconstruction using the Avizo software (version 9.1.1). Scale bar: 500 nm (**b**–**d**), 2 µm (**f**–**h**).

In the high magnification of the proximal areas between two ICs extracted from the 3D image stack (white line square area, inset of Fig. 5a), ICs were in close proximity to each other over a long distance (Fig. 5a). 2D images of the proximal areas between two ICs showed continuous contacts between ICs with the high electron density (black arrows, Fig. 5b–d). In the high-magnification images of areas close to the other two ICs extracted from the 3D image stack (white line square area, inset of Fig. 5e), the two ICs overlapped in a wide range (Fig. 5e). 2D images of the proximal areas between overlapped two ICs also showed that multiple puncta contacts between ICs with high electron density (black arrows, Fig. 5f–h).

**Figure 6:**
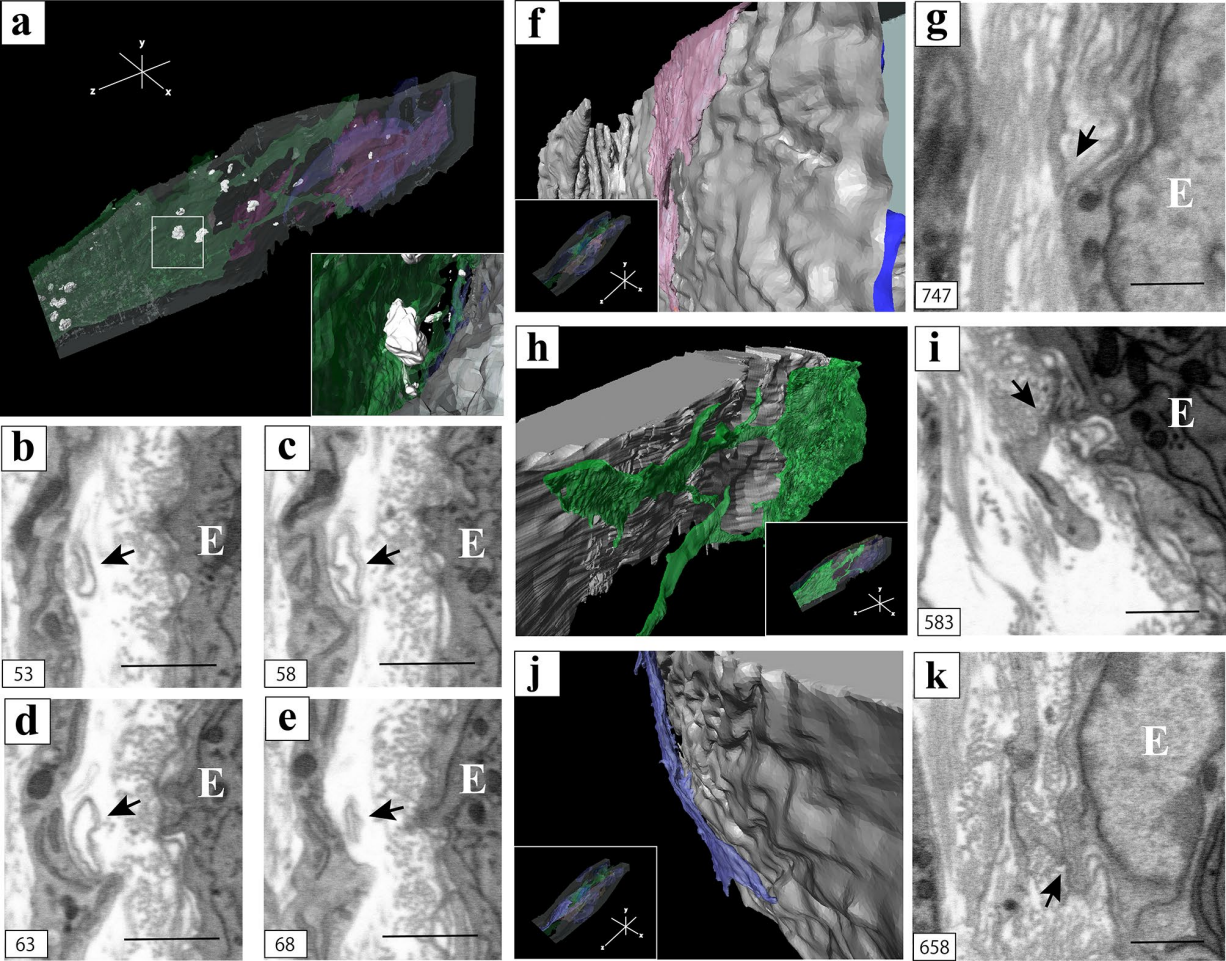
(**a**) 3D reconstruction of multiple extracellular vesicles (EV)-like structures and the interstitial cells (ICs). High magnification of the white square area of (**a**) [inset of (**a**)]; 2D digital slices extracted from the sequential images of the area reconstructed in (**a**). (**b**–**e**) Loop-like structures around the ICs [black arrows of (**b**–**e**)]; 3D reconstruction of the proximity areas between ICs and epithelium. (**f**, **h**, **j**) The spatial relationship between (**f**, **h**, **j**) and the whole 3D reconstruction images [inset of (**f**, **h**, **j**)]; 2D digital slices extracted from the sequential images of the area reconstructed in (**f**, **h**, **j**) (**g**, **i**, **k**); The proximity areas between ICs and epithelium [black arrows of (**g**, **i**, **k**)]. E indicates the epithelium (**b**–**e**, **g**, **i**, **k**). For better visualization, the object surfaces are color-coded; ICs, pink, green and indigo blue; EV-like structures, white; epithelium, gray. The images underwent deconvolution and 3D reconstruction using the Avizo software (version 9.1.1). Scale bar: 500 nm (**b**–**e, g, i, k**).

Multiple extracellular vesicle (EV)-like structures were observed around the IC (Fig. 6a). These EV-like structures were spherical and spheroidal in size and shape (inset of Fig. 6a). In the 2D sequential images, loop-like structures around the ICs were observed (black arrows, Fig. 6b–e). In the high magnification of the close proximity areas between subepithelial ICs and epithelium (inset of Fig. 6f, h, j), subepithelial ICs covered the epithelium (Fig. 6f, h, j). 2D images of the proximal areas between subepithelial ICs and the epithelium showed no contacts between subepithelial ICs and the epithelium with gap junctions (black arrows, Fig. 6g, i, k).

**Figure 7:**
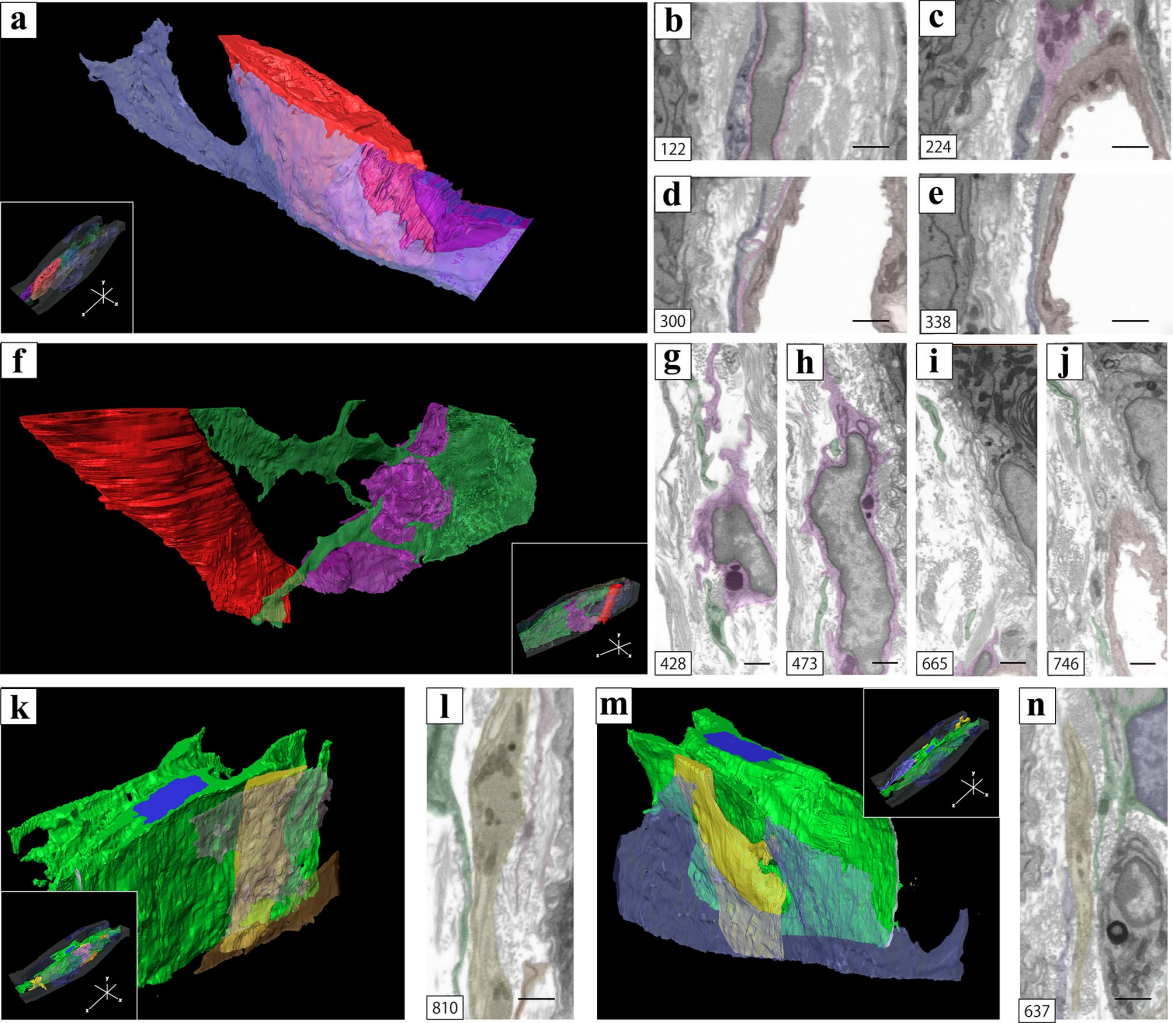
(**a**, **f**) 3D reconstruction of the proximity areas among ICs, macrophages, and vessels. The spatial relationship between (**a**, **f**) and the whole 3D reconstruction images [inset of (**a**, **f**)]. (**b**–**e**, **g**–**j**) 2D digital slices extracted from the sequential images of the area reconstructed in (**a**, **f**). (**k**, **m**) 3D reconstruction of the proximity areas between ICs and nerves. The spatial relationship between (**k**, **m**) and the whole 3D reconstruction images [inset of (**k**, **m**)]. (**l**, **n**) 2D digital slices extracted from the sequential images of the area reconstructed in (**k**, **m**). For better visualization, the object surfaces are color-coded; ICs, indigo blue, green, light green, beige, ocher; macrophages, purple; vessels, red; nerves, yellow; nuclear, blue. The images underwent deconvolution and 3D reconstruction using the Avizo software (version 9.1.1). Scale bar: 500 nm (**b**–**e, g**–**j**, **l**, **m**).

Regarding the spatial relationship between vessels, macrophages, and ICs extracted from the 3D image stack (inset of Fig. 7a, f), the thin cytoplasm of the IC covered a vessel and a macrophage (Fig. 7a). They were noted to be close to each other (Fig. 7b–e); however, cell junctions between the IC and the vessel or the macrophage were not observed (Fig. 7b–d). Moreover, regarding the spatial relationship for the vessel, macrophage, and IC extracted from the other 3D image stack (inset of Fig. 7f), the IC in close proximity to a macrophage extended its cell processes into a vessel (Fig. 7f). In 2D images extracted from sequential images, the isolated cytoplasm of the IC was in close proximity to the macrophage and the vessel, but no cell junctions were observed (Fig. 7g, h, j). The macrophage was also in close proximity to the vessel (Fig. 7i). In the spatial relationship for ICs and nerves extracted from the 3D image stack (inset of Fig. 7k, m), the nerves were sandwiched between ICs (Fig. 7k, m). 2D images showed that the nerve was also in close proximity to the ICs but there were no cell junctions between the IC and the nerve (Fig. 7l, n).

## Discussion

In the present study, we observed the lamina propria of the murine vas deferens at low magnification by CLSM in order to understand an overall morphology or cell arrangement of ICs. Multipolar and bipolar ICs were observed in the lamina propria of the murine vas deferens and they were laid on top of one another in parallel to the epithelium. Furthermore, double staining for PDGFRα and connexin43 suggested that many gap junctions may be present in the cytoplasm of ICs, and double staining for PDGFRα and Iba-1 or β-3tubulin suggested that ICs have contact with not only epithelium but also other surrounding cells. These immunostaining findings were similar to those in previous studies on ICs morphology^26^. However, the finding that ICs were observed like a “wall” is different from findings in previous studies^26^. Previous studies have reported that ICs have a spindle shape, and the present finding suggest that ICs are not spindle-shaped. Since it is difficult to observe more detailed 3D structure by CLSM, we performed FIB/SEM tomography.

In the presented 3D reconstructed images of FIB/SEM, ICs in the lamina propria had a sheet-like structure of cytoplasm. This finding explains that ICs were observed like a “wall” in the 3D image stacks of CLSM and that the 3D structure was similar to that of ICs in the smooth muscle layer of the murine vas deferens^24^. The sheet-like structure of ICs within the lamina propria was parallel to the epithelium, and EV-like structures were observed around the ICs, similar to that in previous reports^26–28^. These results suggest that ICs within the lamina propria exchange humoral factors via EVs. A similar finding was reported in a previous study that observed ICs within the lamina propria of the human bladder using FIB/SEM^29^. EVs secreted by ICs contain mainly proteins, lipids, microRNAs, mRNAs, and mitochondrial DNA (mtDNA), indicating a key role of these cells in intercellular signaling of the interstitial compartment, influencing the function and/or modification of post-transcriptional activity of the surrounding cells^30^ The sheet-like structure of ICs parallel to the epithelium is a very reasonable structure for intercellular communication via EVs because it increases the surface area of the cell membrane to produce more EVs or to express more receptors that bind to EVs^29^.

In the FIB/SEM image stack, ICs were in contact with each other via gap junctions, and this finding is consistent with the CLSM finding. Although cross-sectional 2D SEM images showed small high-electron density areas between ICs, these small high-electron density areas were observed to slide continuously over many sequential 2D images. In the 3D reconstructed images of these sequential 2D images, the ICs were in close proximity to each other over a long distance; these findings suggest the existence of a large gap junction. On the other hand, small high-electron density areas between ICs were observed continuously over a few sequential 2D images, and these findings suggest the existence of adherens junctions. It has been reported that ICs within other organs have an electrical connection via gap junctions and have a signal transduction via adherens junctions^8,31,32^ and the present study had similar findings.

We found morphological evidence that ICs within the lamina propria of the murine vas deferens have gap junctions or adherens junctions between them, as well as the existence of multiple EVs near ICs. This morphological evidence was revealed by 3D analysis using FIB/SEM for the first time. The present study suggests that ICs within the lamina propria of the murine vas deferens formed a complex 3D network via gap junctions and exchange electrical signals. ICs formed homocellular networks by means of gap junctions or adherens junctions, however no such junctions could be seen between ICs and surrounding other cells and tissues. This result reinforced the idea that gap junctions are restricted to homocellular communication.

ICs were in close proximity to not only the epithelium, but also vessels, macrophages, and nerves. Previous studies also suggest that ICs may be involved in nervous, vascular, and immune system functions, as well as regulation of tissue homeostasis and long-distance communication through intercellular signaling by "stromal synapse" because of their strategic position near other cells and tissues^33^. "Stromal synapse" is defined by intercellular distances within the molecular interaction range (15–100 nm). Although no obvious cell junctions were observed between these structures and ICs^32^, ICs within the lamina propria of vas deferens were in close proximity to these structures within the molecular interaction range. Therefore, ICs may also physically interact with not only the epithelium but also surrounding tissues and cells by "stromal synapse."

Some of the ICs in the lamina propria had a multiple cell process with different 3D structures. These cell processes were mainly divided into two types: one was an elongated rod-like cell process and the other was folded cytoplasm like an accordion. The rod-like structure is reasonable for direct contact with surrounding ICs or epithelium. The folded-like structure is reasonable for intercellular communication via EVs due to the increased cell surface area, similar to the sheet-like structure. From these structural rationality of cell processes, two types of cell processes with different 3D structures may reflect the ICs have two types of signal transduction, which are physical interaction and exchanging humoral factors via EVs.

Our study has some limitations. Firstly, ICs have considerably long extensions of over 100 μm, and it is difficult to image the entire cell in an FIB/SEM continuous image stack. Image stacks acquired at a lower magnification make it challenging to observe finer cell projections and intercellular adhesion surfaces. The difficulty is furthered due to the lack of a basement membrane in ICs. It may be more effective to use array tomography for 3D reconstruction of the entire cell for IC imaging. Secondly, it is difficult to accurately integrate immunohistochemical characteristics with 3D observations of the IC ultrastructure using the presented method. This limitation can be overcome using 3D correlative light-electron microscopy^34–36^. Finally, the major limitation of our study is that only a small number of samples could be analyzed by 3D ultrastructure reconstruction, due to the time-consuming manual segmentation. Therefore, we could not quantify the findings and analyze the data.

In conclusion, the present study revealed that in addition to the existence of many EVs surrounding ICs, the contact between ICs and surrounding tissue and cells, including the epithelium, are via gap junctions, adherens junctions, or stromal synapse. From these results, we suggest that ICs physically interact with surrounding tissue and cells, and exchange humoral factors with these structures via EVs. Two types of cell processes with 3D structural differences may reflect that ICs have two types of signal transduction, as mentioned above. ICs within the lamina propria of the murine vas deferens may play an important role in the transduction of signals obtained from the epithelium to the smooth muscles by forming a 3D network. The 3D structure of ICs and their spatial relationship with the epithelium provide new insights into the contraction functions of the vas deferens.

## Supplementary Information

Supplementary Information.

## Data Availability

All data generated or analyzed in this study are included in this article and Supplementary Information Files.

## References

[1] Koslov DS, Andersson KE (2013). Physiological and pharmacological aspects of the vas deferens-an update. Front. Pharmacol..

[2] Marberger H (1974). The mechanisms of ejaculation. Basic Life Sci..

[3] Berridge MJ (2008). Smooth muscle cell calcium activation mechanisms. J. Physiol..

[4] Zhao L (2021). Regulation of smooth muscle contractility by the epithelium in rat tracheas: Role of prostaglandin E2 induced by the neurotransmitter acetylcholine. Ann. Transl. Med..

[5] Ruan YC, Zhou W, Chan HC (2011). Regulation of smooth muscle contraction by the epithelium: Role of prostaglandins. Physiology.

[6] Ruan YC (2008). Regulation of smooth muscle contractility by the epithelium in rat vas deferens: Role of ATP-induced release of PGE2. J. Physiol..

[7] Mirancea N (2016). Telocyte: A particular cell phenotype: Infrastructure, relationships and putative functions. Rom. J. Morphol. Embryol..

[8] Edelstein L, Smythies J (2014). The role of telocytes in morphogenetic bioelectrical signaling: Once more unto the breach. Front. Mol. Neurosci..

[9] Sanders KM, Ward SM, Koh SD (2014). Interstitial cells: regulators of smooth muscle function. Physiol. Rev..

[10] Koh SD, Lee H, Ward SM, Sanders KM (2018). The mystery of the interstitial cells in the urinary bladder. Annu. Rev. Pharmacol. Toxicol..

[11] Radu BM (2017). Calcium signaling in interstitial cells: focus on Telocytes. Int. J. Mol. Sci..

[12] Grainger N (2020). Identification and classification of interstitial cells in the mouse renal pelvis. J. Physiol..

[13] Vannucchi MG, Traini C, Guasti D, Del Popolo G, Faussone-Pellegrini MS (2014). Telocytes subtypes in human urinary bladder. J. Cell. Mol. Med..

[14] Huizinga JD, Zarate N, Farrugia G (2009). Physiology, injury, and recovery of interstitial cells of Cajal: Basic and clinical science. Gastroenterology.

[15] Merrill L, Gonzalez EJ, Girard BM, Vizzard MA (2016). Receptors, channels, and signalling in the urothelial sensory system in the bladder. Nat. Rev. Urol..

[16] Berezin I, Huizinga JD, Daniel EE (1990). Structural characterization of interstitial cells of Cajal in myenteric plexus and muscle layers of canine colon. Can. J. Physiol. Pharmacol..

[17] Komuro T, Seki K, Horiguchi K (1999). Ultrastructural characterization of the interstitial cells of Cajal. Arch. Histol. Cytol..

[18] Popescu LM, Faussone-Pellegrini MS (2010). Telocytes: A case of serendipity: the winding way from interstitial cells of cajal (ICC), via interstitial cajal-like cells (ICLC) to telocytes. J. Cell. Mol. Med..

[19] Koh BH (2012). Platelet-derived growth factor receptor-α cells in mouse urinary bladder: A new class of interstitial cells. J. Cell. Mol. Med..

[20] Kizilyaprak C, Stierhof YD, Humbel BM (2019). Volume microscopy in biology: FIB-SEM tomography. Tissue Cell.

[21] Cretoiu D (2016). The third dimension of telocytes revealed by FIB-SEM tomography. Adv. Exp. Med. Biol..

[22] Ohta K (2012). Beam deceleration for block-face scanning electron microscopy of embedded biological tissue. Micron.

[23] Walton J (1979). Lead aspartate, an en bloc contrast stain particularly useful for ultrastructural enzymology. J. Histochem. Cytochem..

[24] Hiroshige T (2022). Three-dimensional analysis of interstitial cells in the smooth muscle layer of murine vas deferens using confocal laser scanning microscopy and FIB/SEM. Microsc. Microanal..

[25] Murakami M, Nishida T, Iwanaga S, Shiromoto M (1984). Scanning and transmission electron microscopic evidence of epithelial phagocytosis of spermatozoa in the terminal region of the vas deferens of the cat. Experientia.

[26] Hiroshige T (2021). Identification of PDGFRα-positive interstitial cells in the distal segment of the murine vas deferens. Sci. Rep..

[27] Popescu LM, Gherghiceanu M, Cretoiu D, Radu E (2005). The connective connection: Interstitial cells of Cajal (ICC) and ICC-like cells establish synapses with immunoreactive cells: Electron microscope study in situ. J. Cell. Mol. Med..

[28] Bei Y, Zhou Q, Sun Q, Xiao J (2016). Telocytes in cardiac regeneration and repair. Semin. Cell Dev. Biol..

[29] Neuhaus J (2018). 3D-electron microscopic characterization of interstitial cells in the human bladder upper lamina propria. Neurourol. Urodyn..

[30] Yang R, Tang Y, Chen X, Yang Y (2021). Telocytes-derived extracellular vesicles alleviate aortic valve calcification by carrying miR-30b. ESC Heart Fail..

[31] Marini M (2017). Telocytes in normal and keratoconic human cornea: An immunohistochemical and transmission electron microscopy study. J. Cell. Mol. Med..

[32] Popescu LM, Fertig ET, Gherghiceanu M (2016). Reaching out: Junctions between cardiac telocytes and cardiac stem cells in culture. J. Cell. Mol. Med..

[33] Gherghiceanu M, Popescu LM (2012). Cardiac telocytes: Their junctions and functional implications. Cell Tissue Res..

[34] Eckly A, Rinckel JY, Proamer F, Gachet C (2018). High-resolution 3D imaging of megakaryocytes using focused ion beam-scanning electron microscopy. Methods Mol. Biol..

[35] Gemin O (2021). Unique properties of dually innervated dendritic spines in pyramidal neurons of the somatosensory cortex uncovered by 3D correlative light and electron microscopy. PLOS Biol..

[36] Ohta K, Hirashima S, Miyazono Y, Togo A, Nakamura KI (2021). Correlation of organelle dynamics between light microscopic live imaging and electron microscopic 3D architecture using FIB-SEM. Microscopy.

