# Supplementary material for: Three-dimensional analysis of interstitial cells in the lamina propria of the murine vas deferens by confocal laser scanning microscopy and FIB/SEM
Source: Sci Rep. 2022 Jun 8;12:9484. doi: 10.1038/s41598-022-13245-7 (PMC9177838; doi:10.1038/s41598-022-13245-7)
Supplement: Supplementary file 1 — Supplementary Information. [file 41598_2022_13245_MOESM1_ESM.pdf]

Title: Three-dimensional analysis of interstitial cells in the lamina propria of the murine vas deferens by confocal laser scanning microscopy and FIB/SEM

TASUKU HIROSHIGE[1*], KEI-ICHIRO UEMURA[1], SHINGO HIRASHIMA[2], AKINOBU TOGO[3], KEISUKE OHTA[2, 3], KEI-ICHIRO NAKAMURA[4] and TSUKASA IGAWA[1]

[1] Department of Urology, Kurume University School of Medicine, Kurume, 830-0011, Japan; [2] Division Microscopic and Development Anatomy, Department of Anatomy, Kurume University School of Medicine, Kurume, 830-0011, Japan; [3] Advanced Imaging Research Center, Kurume University School of Medicine, Kurume, 830-0011, Japan; [4] Cognitive and Molecular Research Institute of Brain Diseases, Kurume University School of Medicine, Kurume 830-0011, Japan.

**Correspondence to*: Dr. Tasuku Hiroshige, Department of Urology Kurume University School of Medicine, Kurume, 830-0011, Japan.

E-mail: hiroshige_tasuku@kurume-u.ac.jp

Key words: three-dimensional analysis, interstitial cells, FIB / SEM, murine vas deferens

Running title: HIROSHIGE *et al*: Three-dimensional analysis of interstitial cells in the lamina propria of the murine vas deferens

Supplementary Figure S1: 2D digital slice extracted from the sequential immunofluorescence images for platelet-derived growth factor α (PDGFRα) (green) and Iba-1 (red).

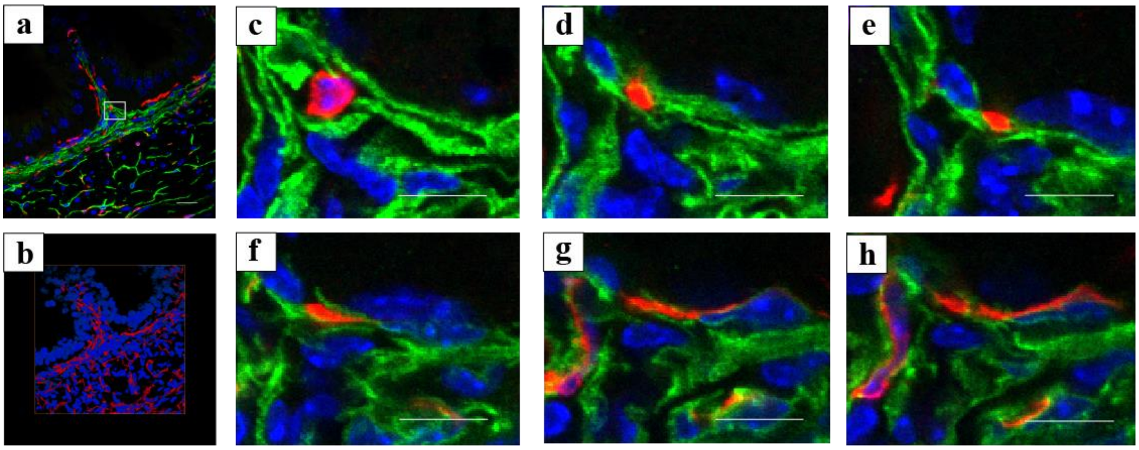

(**a**) Reconstructed sequential 3D immunofluorescence images for Iba-1 (red). (**b**) Sequential high-magnification immunofluorescence images of frozen sections for PDGFRα (green) and Iba-1 (red). (**c-j**) The images underwent deconvolution and 3D reconstruction using the Avizo software (version 9.1.1).

Scale bar: 20 μm (**c-j**).

Supplementary Figure S2: 2D digital slice extracted from the sequential immunofluorescence images for platelet-derived growth factor α (PDGFRα) (green) and β3-tubulin (red).

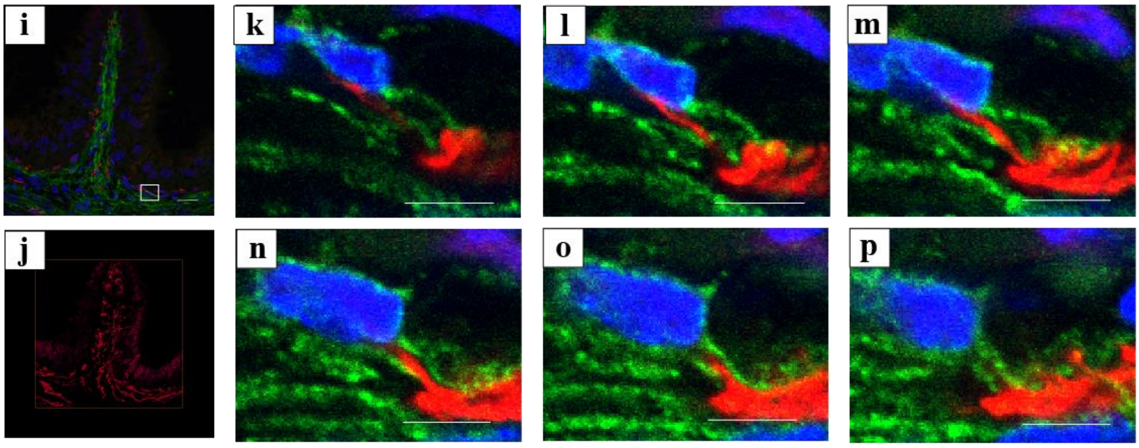

(**a**) Reconstructed sequential 2D digital slice immunofluorescence images for PDGFRα (green) and β3-tubulin (red). (**b**) Sequential high-magnification immunofluorescence images of frozen sections for PDGFRα (green) and β3-tubulin (red). (**d-k**) The same images underwent deconvolution and 3D reconstruction using the Avizo software (version 9.1.1).

Scale bar: 20 μm (**c-j**).

Supplementary Table S1

**Primary antibody**	**Host**	**Working dilution**	**Source**
PDGFR-α	Goat	1:400	Catalog no. AF1062; R&D systems
β3-tublin	Rabbit	1:500	Catalog no. ab18207; abcam
Iba-1	Rabbit	1:250	Catalog no. 019-19741; wako
connexin43	Rabbit	1:1000	Catalog no. ab34710; abcam

Details of the primary antibodies
